# Development and Validation of an Undiagnosed Diabetes Screening Tool: Based on the Korean National Health and Nutrition Examination Survey (2010–2016)

**DOI:** 10.3390/healthcare9091138

**Published:** 2021-08-31

**Authors:** Eunhee Cho, Deulle Min, Hye Sun Lee

**Affiliations:** 1 Mo-Im Kim Nursing Research Institute, Yonsei University College of Nursing, Seoul 03722, Korea; ehcho@yuhs.ac; 2 Department of Nursing, College of Medicine, Wonkwang University, Iksan 54538, Korea; 3 Biostatistics Collaboration Unit, Yonsei University College of Medicine, Seoul 06229, Korea; hslee1@yuhs.ac

**Keywords:** diabetes early diagnosis, health check-ups, screening tool, smoking cessation, undiagnosed diabetes

## Abstract

Approximately half of the population worldwide suffers from under/undiagnosed diabetes. In South Korea, 27.7% of people aged over 30 years have type 2 diabetes and are unaware of their condition because they have not been diagnosed. Optimal tools for identifying risk factors of undiagnosed diabetes, which is associated with multiple complications, are currently lacking. Secondary data analysis was conducted using the 2010–2016 Korean National Health and Nutrition Examination Survey. This study aimed to identify the risk factors in individuals not diagnosed with type 2 diabetes, using glycated hemoglobin as the diagnostic standard. Furthermore, we aimed to develop an accurate screening tool for diabetes using HbA1c values by analyzing the data of 12,843 adults (aged ≥20 years) not diagnosed with type 2 diabetes. Age, gender, family history of diabetes, hypertension diagnosis, waist-to-height ratio, smoking, and health check-ups were identified as significant risk factors for undiagnosed type 2 diabetes. A screening tool with total and cutoff scores of 13 and 7 points was developed, and it had a sensitivity of 82.7% and specificity of 58.2%. The developed screening tool appears to be a simple and cost-effective method for detecting undiagnosed type 2 diabetes.

## 1. Introduction

It is estimated that 225 million or 50% of 20- to 79-year-olds with type 2 diabetes worldwide are unaware of their disease [1]. The number of patients with type 2 diabetes has increased in South Korea. As of 2016, there were approximately 5 million (14.4%) patients with type 2 diabetes aged over 30 years; among them, approximately 30% were ≥65 years [2]. In South Korea, 27.7% of people aged over 30 years with type 2 diabetes are unaware of their condition because they have not been diagnosed [3].

In South Korea, free health check-ups for citizens aged ≥40 years are provided once every 2 years to improve disease prevention and healthcare. Moreover, individuals receive additional health screenings if necessary [4]. Type 2 diabetes screening involves a basic blood test, followed by a second test for individuals suspected to have diabetes. In 2017, the Korean health screening rate was 78.5%, representing a 5.6% increase since 2012 (72.9%) [4]. In other words, 21.5% of eligible people do not undergo health check-ups.

In general, a person who does not undergo health check-ups has a low economic status, health value, self-efficacy, health control, and apprehensions about the effectiveness of the health check-ups [5]. According to the health belief model, people may not undergo health check-ups because they underestimate their susceptibility to diabetes and disease severity [6]. Underdiagnosed or poorly managed diabetes is associated with complications such as retinopathy, blindness, limb amputation, end-stage renal failure, and cardiovascular disease, all of which can increase medical costs and chances of mortality [1,7,8].

Early detection of undiagnosed type 2 diabetes can reduce subsequent complications and prevent deaths. However, diagnosing diabetes can be very challenging for the patient and the medical staff as many tests are required [9]. Many countries have provided tools to assess the risk of type 2 diabetes in order to detect it early [10,11]. However, there are differences in the sensitivity and specificity of diabetes screening.

The American Diabetes Association (2017) recommended glycated hemoglobin (HbA1c) as the new diagnostic criterion in 2009 because the HbA1c test uses a blood sample and has several advantages [9]. It does not require prior fasting and better indicates overall glycemic exposure and risk of long-term complications. It also has greater pre-analytical stability and is less affected by stress or infection than other parameters [9,12]. However, it is inconvenient for an individual to visit a medical institution for examination and bear the healthcare cost. The screening tools in the easy-to-use scoring system help detect undiagnosed diabetes in vulnerable people who live in areas with limited access to healthcare or who cannot make time for hospital visits, such as those who have to care for dependents or have to work every day. This study aimed to develop an accurate screening tool without the need for visiting a medical institution. Further, it aimed to prove the tool’s effectiveness by identifying Korean individuals who had not been diagnosed with type 2 diabetes.

## 2. Materials and Methods

### 2.1. Undiagnosed Diabetes Screening Tool Development

This study comprised a three-step process to develop the tool. In the first step, we screened individuals with undiagnosed diabetes who were not diagnosed with type 2 diabetes by a doctor. For this, we used data from the 2010–2016 Korean National Health and Nutrition Examination Survey (KNHANES) conducted by the Korea Disease Control and Prevention Agency. KNHANES is a cross-sectional survey, which classifies surveyed populations of specific regions and residences according to administrative law and applies a systematic extraction method (stratified cluster sampling). In KNHANES V (2010–2012), 25,534 people in 576 survey districts were surveyed, and in KNHANES VI (2013–2015), 22,948 people in 576 survey districts were surveyed. In KNHANES VII-1wave (2016), 8150 people from 4416 households in 192 survey districts were surveyed. We divided the 2010–2016 data into two parts. Data from 2010–2013 were used to construct the tool, and data from 2014–2016 were used for validation. The dataset from 2010–2013 included 25,422 individuals aged ≥20 years, of whom 21,336 were not diagnosed with type 2 diabetes, 1989 were diagnosed with type 2 diabetes, and 2097 who failed to respond were excluded from the study. Further, 6546 individuals with no available HbA1c data and 1947 individuals with missing key variables were excluded. Finally, the participants used for developing the tool included 12,843 adults aged ≥20 years who had not been diagnosed with type 2 diabetes.

In the second step, we divided participants into normal (HbA1c <6.5%) and undiagnosed (HbA1c ≥6.5%) groups based on HbA1c levels [9]. Thereafter, we constructed an initial tool by identifying factors affecting HbA1c elevation.

Finally, in the third step, to validate the screening tool, data of 23,080 individuals who participated in the 2014–2016 KNHANES, including 18,067 adults aged >20 years, were examined. After excluding those with missing values for any of the variables, 13,938 eligible individuals were included in the validation study. The sensitivity and specificity were examined by applying them to other populations to confirm the tool’s validity. The raw data were made publicly available on the Korean National Health and Nutrition Survey website (https://knhanes.kdca.go.kr, accessed on 18 September2017) [13]. The participants could not be identified because only non-identifiable data were provided. Written informed consent was obtained from all participants before the survey began [13]. This study was conducted after obtaining approval from the institutional review board (approval number: Y-2019-0064).

### 2.2. Variables for Initial Item

HbA1c tests were performed for blood sampling using 21- or 23-gauge needles in the median cubital veins and the cephalic vein (approximately 3 cc), respectively. Blood was collected in EDTA tubes and mixed with an anticoagulant for 10 min using a roller mixer. Blood samples were then stored in a refrigerator at 2–8 °C. The samples were subsequently transferred to a central analysis agency. They were analyzed within 24 h after sampling. HbA1c tests were performed using dedicated reagents (HLC-723 G8, Tosoh Bioscience, Inc., Tokyo, Japan).

The following demographic characteristics were extracted from the datasets: age, which was classified into four groups (<40, 40–49, 50–59, and >60 years), sex (male and female), family history of diabetes (“Yes” if parents and/or siblings had diabetes), and history of hypertension diagnosis (“Yes” if hypertension was diagnosed previously). Anthropometric parameters were measured using a stadiometer (seca 225; seca, Hamburg, Germany), a weighing scale (GL-6000-20; G-tech, Gyeonggi-do, Korea), and a waist circumference (WC) ruler (seca 200; seca). WC was measured (in cm) at the mid-axillary line, defined as the midpoint between the two points identified by palpating the lower end of the last rib and the two upper points of the iliac crest. Participants were classified into three sex-stratified groups according to WC (male: <84.0 cm, 84.0–89.9 cm, and ≥90 cm; female: <77.0 cm, 77.0–83.9 cm, and ≥84 cm) [14]. The waist-to-height ratio (WHR) was calculated as the ratio of the WC (cm) to height (cm) and used to classify participants into two groups (≤0.5 and ≥0.5) [15]. Body mass index (BMI) was calculated as weight (kg) divided by the square of height (m^2^). It was used to classify participants into four groups (<23, 23–24.9, 25–29.9, and ≥30 kg/m^2^) based on the World Health Organization’s criteria for Asian populations [16].

The lifestyle and health behavioral characteristics included the total daily energy intake (kcal), frequency of eating out, physical activity, smoking, drinking, and participation in regular health screenings. For men and women (aged 30–60 years), the respective total daily energy intakes were classified as <2300 and 1850 kcal or ≥2300 and 1850 kcal [17]. Eating out was dichotomized as “Yes” or “No.” Physical activity was recorded as “Yes” if intensive physical activity was performed for ≥20 min, ≥3 days/week, or moderate physical activity was performed for ≥30 min, ≥5 days/week. Smokers were categorized as present smokers or non/past-smokers. Drinking was recorded as “Yes” if more than 1 drinking episode/month was reported. Participation in health screenings was recorded as “Yes” if the participant had undergone a health check-up within the last 2 years.

### 2.3. Data Analysis

Data analysis was performed using the PASW SPSS Statistics for Windows, version 24.0 (IBM Corp., Armonk, NY, USA), and MedCalc for Windows, version 18.0, software packages (MedCalc Software, Mariakerke, Belgium). A descriptive analysis of the characteristics of the two groups was performed, and differences between the groups were analyzed using the χ^2^ test. The Delong method was used for comparative analysis of the areas under the receiver operating characteristic curves (AUC) between risk variables. Risk factors were extracted using multiple logistic regression analysis, and risk scores were assigned according to the odds ratios (ORs). Youden’s Index J (Sensitivity + Specificity-1) was used to determine the cutoff scores of the newly developed screening tool.

## 3. Results

### 3.1. General Characteristics of the Subjects

The mean ages of participants in the normal HbA1c (<6.5%) and undiagnosed groups were 49.0 and 59.2 years, respectively. Older people were found in the undiagnosed group (χ^2^ = 236.63, *p* < 0.001). The normal group included a larger proportion of women (χ^2^ = 8.99, *p* = 0.003). The prevalence of family history of diabetes was 5.8% higher in the undiagnosed group than in the normal group (χ^2^ = 13.98, *p* < 0.001). A history of hypertension was 22.4% higher in the undiagnosed group than in the normal group (χ^2^ = 189.04, *p* < 0.001). In the undiagnosed group, all three indices of obesity (BMI: χ^2^ = 345.09, *p* < 0.001; WC: χ^2^ = 355.69, *p* < 0.001; WHR: χ^2^ = 325.13, *p* < 0.001) were higher than the normal group. Furthermore, 66.0% of participants in the undiagnosed group had a total energy intake ≤2300/1850 kcal/day, which was higher than the normal group (χ^2^ = 9.60, *p* = 0.002). The proportion of participants who reported “No eating out” was 13% higher, and the proportion of drinkers was lower (χ^2^ = 9.24, *p* = 0.002) in the undiagnosed group compared to the normal group (χ^2^ = 45.16, *p* < 0.001; Table 1).

### 3.2. Discriminatory Ability of HbA1c in BMI, WC, and WHR

The AUCs of BMI, WC, and WHR were compared to determine the best indicator of type 2 diabetes. WHR was confirmed to be a better indicator than BMI or WC, and a comparison of the AUCs revealed differences of 0.044 (*p* < 0.001) and 0.014 (*p* = 0.006), respectively.

### 3.3. Risk Factors for Undiagnosed Type 2 Diabetes and Screening Scores

A multiple logistic regression analysis, which was used to determine the factors affecting undiagnosed diabetes, identified age, gender, family history of diabetes, diagnosis of hypertension, WHR, smoking, and participation in health screening as statistically significant. Specifically, the risk of undiagnosed diabetes was 3.03 times higher among those aged 40–49 years (OR: 3.03, 95% confidence interval (CI): 2.094–4.373), 4.30 times higher among those aged 50–59 years (OR: 4.30, 95% CI: 3.023–6.122), and 5.06 times higher among those aged ≥60 years (OR: 5.06, 95% CI: 3.569–7.159) compared to those aged <40 years. Men had a 1.26 times higher risk of undiagnosed type 2 diabetes compared to women (OR: 1.26, 95% CI: 1.047–1.516). Those with a family history of diabetes had a 1.92 times higher risk of undiagnosed type 2 diabetes than those without (OR: 1.92, 95% CI: 1.571–2.369). History of diagnosed hypertension was associated with a 1.58 times higher risk of undiagnosed type 2 diabetes (OR: 1.58, 95% CI: 1.314–1.906), while a WHR ≥0.5 increased the risk of undiagnosed diabetes by 3.75 times (OR: 3.75, 95% CI: 3.025–4.669). Additionally, smoking was associated with a 1.40 times increase in the risk of undiagnosed type 2 diabetes (OR: 1.40, 95% CI: 1.113–1.767). Irregular participation in health screening check-ups had a 1.46 times higher risk of undiagnosed type 2 diabetes (OR: 1.46, 95% CI: 1.229–1.739). The risk included in the new screening tool was reported according to the OR. This tool yielded a maximum score of 13 points, with a risk cutoff of 7 points. Its sensitivity and specificity were 82.7% and 58.2%, respectively (Table 2). The sensitivity and specificity of the newly developed undiagnosed diabetes screening tool were higher (2.4% and 0.8%, respectively) than those of a previously developed tool, which examined risk factors of diabetes in the Korean population [14] (Table 3).

### 3.4. Validity Analysis

The 2014–2016 KNHANES data were used to validate the newly developed tool. The average age of the participants in this survey of 13,938 adults aged ≥20 years was 49.72, and 42.6% were men. Undiagnosed diabetes was detected in 453 people (3.3%). According to this tool, the AUC was 0.726, with 82.3% sensitivity and 53.8% specificity.

## 4. Discussion

In this study, we developed a screening tool to identify the risk of undiagnosed type 2 diabetes in adults based on HbA1c as the criterion for diagnosing diabetes. We identified 7 factors (age, gender, family history of diabetes, diagnosis of hypertension, WHR, smoking, and participation in health screening), which were significant predictors of an individual’s risk for developing undiagnosed type 2 diabetes. These factors are different from the existing scale developed for the risk of type 2 diabetes [14]. Our results showed that the tool (with improved sensitivity and specificity) developed in this study more accurately detected undiagnosed diabetics than simply an increase in number. We believe this difference is due to the use of HbA1c as the diagnostic criterion for type 2 diabetes. HbA1c usually reflects the average blood glucose level over the previous three months, but not the blood glucose level at the time of the blood test [12,18].

In our previous study, sex was not a risk factor for undiagnosed diabetes. However, this study result showed that men were at a higher risk of undiagnosed diabetes than women [19]. This is consistent with previous studies, which showed that men are at a higher risk of diabetes than women [10]. Further research is needed to find out why men are undiagnosed more often.

We investigated the relationship between several obesity indicators that best represent the risk of type 2 diabetes in the current Korean population. Our results demonstrated that WHR was a better predictor of type 2 diabetes than BMI and WC. This was consistent with previous studies that reported WHR as a predictor of cardiovascular disease due to the high distribution of visceral fat in the Asian population, despite low BMI or WC [15,20]. A WHO report also supported this finding and stated that as the fat distributions differ between individuals in Asian and Western countries, the criteria used to categorize BMI should also differ [16]. Another study found that when only the WC was considered, individuals with the same WC cutoff points but different heights could have different levels of risk. Accordingly, both height and WC should be considered [21]. Although BMI has been used as a representative marker of obesity by Korea and the WHO, the unit for height must be changed from centimeters to meters before calculation, which is challenging. In contrast, the WHR involves only a simple calculation that does not require a calculator. However, further studies are needed to determine whether WHR is an appropriate indicator of obesity in other countries.

We identified that participants’ lifestyle and health-related behavioral factors in regular health screening check-ups and smoking were significant factors associated with the undiagnosed type 2 diabetes risk. Interest in health promotion and disease prevention increases health screening check-ups, especially as the population ages and chronic disease burdens increase [22]. This study revealed that regular participation in health screening check-ups was a better indicator of the risk of developing undiagnosed type 2 diabetes than the total daily calorie intake, eating out, drinking, or physical activity. Participation in health check-ups is a positive and effective approach for disease prevention, which enables the diagnosis of a new disease even if there is no reduction of the related incidence and mortality [23,24]. Therefore, it is necessary to emphasize the importance of health screening check-ups, identify the underlying reasons why some people do not receive further screening, and develop appropriate health promotions intended to increase participation in health screenings among the general population. This study also identified smoking as a strong risk factor, which was consistent with the inclusion of smoking in other screening tools [14,25]. Further, it was found that smoking increases the risk of type 2 diabetes and also increases mortality [26]. This screening tool could facilitate the early treatment of patients with a high risk of undiagnosed diabetes.

This study had some limitations. First, only KNHANES participants who had not been diagnosed with type 2 diabetes and had the main variables available were included. Therefore, generalizing the results for all individuals who were not diagnosed with type 2 diabetes may be limited. For further generalization, a large-scale study of people who have not been diagnosed with diabetes is needed. Second, the total daily energy intake was based on the daily recommended values for Korean men and women and did not account for the types of foods actually consumed. Moreover, all questionnaires were self-reported, and a secondary analysis was performed. Eating out was dichotomized as “Yes” or “No,” which limited the accuracy because the number of events and types of food were not considered. Therefore, additional research that includes the types of food consumed per day is warranted. Third, previous studies showed that low consumption of fruits and vegetables, overeating per day, and low physical activity are risk factors for diabetes and obesity [27,28]. Drinking and physical activity were not included in the tool we developed. In a previous study examining the relationship between health behavior, obesity, and diabetes, drinking and exercise were not related factors [29]. Future studies of detailed dietary records are needed to elucidate the relationships among the total energy intake, dietary content, obesity, and risk of undiagnosed type 2 diabetes. Lastly, this was a tool development study using secondary data, and there was limited scope for carrying out all the steps of tool development suggested in the existing literature (expert panel review, etc.) [30]. Nevertheless, this study is meaningful because it developed a screening tool based on the actual data of subjects with undiagnosed diabetes and confirmed its validity.

## 5. Conclusions

In conclusion, the tool developed in this study includes various known risk factors and may facilitate early detection and treatment of undiagnosed type 2 diabetes in the Korean population. It may be useful for community nurses who specialize in identifying those prone to developing the disease.

## Figures and Tables

**Table 1:** General characteristics of participants (*N* = 12,843).

Variables	Normal	Undiagnosed	χ^2^ Test	*p*-Value
	(*N* = 12,219)	(*N* = 624)		
	*N* (%) or M ± SD	*N* (%) or M ± SD		
Demographic characteristics				
Age (years)	49.34 ± 15.89	59.16 ± 12.55	236.63	<0.001
<40	3857 (31.6)	44 (7.1)		
40–49	2397 (19.6)	96 (15.4)		
50–59	2410 (19.7)	159 (25.5)		
≥60	3555 (29.1)	325 (52.1)		
Gender			8.99	0.003
Women	7675 (60.4)	339 (54.3)		
Men	4844 (39.6)	285 (45.7)		
Family history of DM			13.98	<0.001
No	10,164 (83.2)	483 (77.4)		
Yes	2055 (16.8)	141 (22.6)		
Diagnosis of HTN			189.04	<0.001
No	9961 (81.5)	369 (59.1)		
Yes	2258 (18.5)	255 (40.9)		
Anthropometric characteristics				
Body mass index (kg/m^2^)			345.09	<0.001
<23	5687 (46.6)	114 (18.3)		
23–24.9	2873 (23.5)	137 (22.0)		
25–29.9	3256 (26.6)	291 (46.6)		
≥30	401 (3.3)	82 (13.1)		
Waist circumference (M/W) (cm)			355.69	<0.001
<84/77	6167 (50.5)	115 (18.4)		
84–89.9/77–83.9	3089 (25.3)	160 (25.6)		
≥90/74	2963 (24.2)	349 (55.9)		
Waist-to-height ratio			325.13	<0.001
<0.5	6687 (54.7)	111 (17.8)		
≥0.5	5532 (45.3)	513 (82.2)		
Lifestyle, health behavior characteristics				
Energy intake (M/W) (kcal)			9.60	0.002
≤2300/1850	7307 (59.8)	412 (66.0)		
>2300/1850	4912 (40.2)	212 (34.0)		
Eating out			45.16	<0.001
No	3939 (32.2)	282 (45.2)		
Yes	8280 (67.8)	342 (54.8)		
Physically active			2.12	0.145
No	10,020 (82.0)	526 (84.3)		
Yes	2199 (18.0)	98 (15.7)		
Smokers			3.39	0.066
Non- or past-smokers	10,010 (81.9)	493 (79.0)		
Present smokers	2209 (18.1)	131 (21.0)		
Drinkers			9.24	0.002
Non-drinkers or less than 1 drink per month	5779 (47.3)	334 (53.5)		
More than one drink per month	6440 (52.7)	290 (46.5)		
Health check-ups			0.12	0.725
No	4653 (38.1)	242 (38.8)		
Yes	7566 (61.9)	382 (61.2)		

Note. HbA1c = glycated hemoglobin; *N* = number, M = mean; SD = standard deviation; DM = diabetes mellitus; HTN = hypertension; M/W = men/women.

**Table 2:** Factors affecting HbA1c levels and imputation of scores according to odds ratio.

Variables	Odds Ratio	95% CI	*p*-Value	Risk Score
Age (years) (ref: <40)				0
40–49	3.03	2.094–4.373	<0.001	3
50–59	4.30	3.023–6.122	<0.001	4
≥60	5.06	3.569–7.159	<0.001	5
Gender (ref: Women)				0
Men	1.26	1.047–1.516	0.014	1
Family history of DM (ref: No)				0
Yes	1.92	1.571–2.369	<0.001	1
Diagnosis with HTN (ref: No)				0
Yes	1.58	1.314–1.906	<0.001	1
Waist-to-height ratio (ref: <0.5)				0
≥0.5	3.75	3.025–4.669	<0.001	3
Smoking (ref: No)				0
Yes	1.40	1.113–1.767	0.004	1
Check-ups (ref: Yes)				0
No	1.46	1.229–1.739	<0.001	1

Note. HbA1c = glycated hemoglobin; CI = confidence interval; DM = diabetes mellitus; HTN = hypertension; ref. = reference.

**Table 3:** Comparison of the newly developed undiagnosed diabetes screening tool and existing diabetes screening tools.

Measure	Undiagnosed Diabetes Screening Tool	95% CI	Diabetes Screening Tool [14]	95% CI
Sensitivity	82.7	79.5–85.6	80.3	76.9–83.3
Specificity	58.2	57.3–59.1	57.4	56.6–58.3
AUC	0.760	0.752–0.767	0.740	0.732–0.747

Note. CI = confidence interval; AUC = area under the curve.

## Data Availability

The raw, anonymized data are publicly available on the Korean National Health and Nutrition Survey website (https://knhanes.kdca.go.kr; accessed on 17 September 2017).

## References

[1] International Diabetes Federation IDF Diabetes Atlas, 8nd ed.; 2017. https://www.idf.org/e-library/epidemiology-research/diabetes-atlas/134-idf-diabetes-atlas-8th-edition.html.

[2] Korean Diabetes Association Diabetes Fact Sheet in Korea 2018. http://www.diabetes.or.kr/pro/news/admin.php?mode=list&category=A.

[3] Korea Centers for Disease Control & Prevention (2017). 2017 National Health Statistics. KCDC. https://knhanes.kdca.go.kr/knhanes/main.do.

[4] National Health Insurance Service (2017). 2017 National Health Screening Statistical Yearbook. https://www.nhis.or.kr/nhis/together/wbhaec07000m01.do?mode=view&articleNo=127944&article.offset=0&articleLimit=10.

[5] Dryden R., Williams B., McCowan C., Themessl-Huber M. (2012). What Do We Know About Who Does and Does Not Attend General Health Checks?. Findings From a Narrative Scoping Review. BMC Public Health.

[6] Rosenstock I.M. (1974). Historical Origins of the Health Belief Model. Health Educ. Monogr..

[7] Office of Disease Prevention and Health Promotion Diabetes; 2020. https://www.healthypeople.gov/2020/topics-objectives/topic/diabetes.

[8] Tancredi M., Rosengren A., Svensson A.M., Kosiborod M., Pivodic A., Gudbjörnsdottir S., Wedel H., Clements M., Dahlqvist S., Lind M. (2015). Excess Mortality Among Persons With type 2 Diabetes. N. Engl. J. Med..

[9] American Diabetes Association (2017). 2. Classification and Diagnosis of Diabetes. Diabetes Care.

[10] Bang H., Edwards A.M., Bomback A.S., Ballantyne C.M., Brillon D., Callahan M.A., Teutsch S.M., Mushlin A.I., Kern L.M. (2009). Development and Validation of a Patient Self-Assessment Score for Diabetes Risk. Ann. Intern. Med..

[11] Xin Z., Yuan J., Hua L., Ma Y.H., Zhao L., Lu Y., Yang J.K. (2010). A Simple Tool Detected Diabetes and Prediabetes in Rural Chinese. J. Clin. Epidemiol..

[12] The International Expert Committee (2009). International Expert Committee Report on the Role of the A1C Assay in the Diagnosis of Diabetes. Diabetes Care.

[13] Korea Centers for Disease Control & Prevention Korean National Health and Nutrition Examination Survey. https://knhanes.cdc.go.kr/knhanes/main.do.

[14] Lee Y.H., Bang H., Kim H.C., Kim H.M., Park S.W., Kim D.J. (2012). A Simple Screening Score for Diabetes for the Korean Population: Development, Validation, and Comparison With Other Scores. Diabetes Care.

[15] Browning L.M., Hsieh S.D., Ashwell M. (2010). A Systematic Review of Waist-to-Height Ratio as a Screening Tool for the Prediction of Cardiovascular Disease and Diabetes: 0.5 Could Be a Suitable Global Boundary Value. Nutr. Res. Rev..

[16] WHO Expert Consultation (2004). Appropriate body-mass index for Asian populations and its implications for policy and intervention strategies. Lancet.

[17] Ministry of Health & Welfare (2015). Dietary Reference Intake for Korean 2015. http://www.mohw.go.kr/react/jb/sjb030301vw.jsp?PAR_MENU_ID=03&MENU_ID=032901&CONT_SEQ=337356.

[18] Saudek C.D., Brick J.C. (2009). The Clinical Use of Hemoglobin A1c. J. Diabetes Sci. Technol..

[19] Min D., Cho E. (2019). Risk Factors for Underdiagnosis of Diabetes Based on the Korean National Health and Nutrition Examination Survey 2013–2015. Asia Pac. J. Public Health.

[20] Park S.H., Choi S.J., Lee K.S., Park H.Y. (2009). Waist Circumference and Waist-to-Height Ratio as Predictors of Cardiovascular Disease Risk in Korean Adults. Circ. J..

[21] Schneider H.J., Klotsche J., Silber S., Stalla G.K., Wittchen H.U. (2011). Measuring Abdominal Obesity: Effects of Height on Distribution of Cardiometabolic Risk Factors Risk Using Waist Circumference and Waist-to-Height Ratio. Diabetes Care.

[22] Shim M., Kelly B., Hornik R. (2006). Cancer Information Scanning and Seeking Behavior Is Associated With Knowledge, Lifestyle Choices, and Screening. J. Health Commun..

[23] Sox H.C. (2013). The Health Checkup: Was It Ever Effective? Could It Be Effective?. JAMA.

[24] Krogsbøll L.T., Jørgensen K.J., Larsen C.G., Gøtzsche P.C. (2012). General Health Checks in Adults for Reducing Morbidity and Mortality From Disease: Cochrane Systematic Review and Meta-Analysis. BMJ.

[25] Engelgau M.M., Narayan K.M., Herman W.H. (2000). Screening for type 2 Diabetes. Diabetes Care.

[26] Prospective Studies Collaboration (2009). Body-Mass Index and Cause-Specific Mortality in 900 000 Adults: Collaborative Analyses of 57 Prospective Studies. Lancet.

[27] Lindström J., Tuomilehto J. (2003). The Diabetes Risk Score: A Practical Tool to Predict type 2 Diabetes Risk. Diabetes Care.

[28] Phelan S.M., Burgess D.J., Burke S.E., Przedworski J.M., Dovidio J.F., Hardeman R., Morris M., van Ryn M. (2015). Beliefs About the Causes of Obesity in a National Sample of 4th Year Medical Students. Patient Educ. Couns..

[29] Min D., Cho E. (2018). Associations Among Health Behaviors, Body Mass Index, Hypertension, and Diabetes Mellitus: A Path Analysis. Medicine.

[30] DeVellis R.F. (2016). Scale Development: Theory and Applications.

